# Preparation and performance of random- and oriented-fiber membranes with core–shell structures *via* coaxial electrospinning

**DOI:** 10.3389/fbioe.2022.1114034

**Published:** 2023-01-09

**Authors:** Yunhuan Li, Dalai Jin, Yongyong Fan, Kuihua Zhang, Tao Yang, Chengyu Zou, Anlin Yin

**Affiliations:** ^1^ Department of Materials Engineering, College of Materials and Textiles, Zhejiang Sci-Tech University, Hangzhou, China; ^2^ Key Laboratory of Yarn Materials Forming and Composite Processing Technology, College of Material and Textile Engineering, Jiaxing University, Jiaxing, Zhejiang, China

**Keywords:** coaxial electrospinning, oriented fiber, poly (L-lactide-co-ε-caprolactone), sodium tanshinone II A sulfonate, vascular graft

## Abstract

The cells and tissue in the human body are orderly and directionally arranged, and constructing an ideal biomimetic extracellular matrix is still a major problem to be solved in tissue engineering. In the field of the bioresorbable vascular grafts, the long-term functional prognosis requires that cells first migrate and grow along the physiological arrangement direction of the vessel itself. Moreover, the graft is required to promote the formation of neointima and the development of the vessel walls while ensuring that the whole repair process does not form a thrombus. In this study, poly (l-lactide-co-ε-caprolactone) (PLCL) shell layers and polyethylene oxide (PEO) core layers with different microstructures and loaded with sodium tanshinone IIA sulfonate (STS) were prepared by coaxial electrospinning. The mechanical properties proved that the fiber membranes had good mechanical support, higher than that of the human aorta, as well as great suture retention strengths. The hydrophilicity of the oriented-fiber membranes was greatly improved compared with that of the random-fiber membranes. Furthermore, we investigated the biocompatibility and hemocompatibility of different functional fiber membranes, and the results showed that the oriented-fiber membranes containing sodium tanshinone IIA sulfonate had an excellent antiplatelet adhesion effect compared to other fiber membranes. Cytological analysis confirmed that the functional fiber membranes were non-cytotoxic and had significant cell proliferation capacities. The oriented-fiber membranes induced cell growth along the orientation direction. Degradation tests showed that the pH variation range had little change, the material mass was gradually reduced, and the fiber morphology was slowly destroyed. Thus, results indicated the degradation rate of the oriented-fiber graft likely is suitable for the process of new tissue regeneration, while the random-fiber graft with a low degradation rate may cause the material to reside in the tissue for too long, which would impede new tissue reconstitution. In summary, the oriented-functional-fiber membranes possessing core–shell structures with sodium tanshinone IIA sulfonate/polyethylene oxide loading could be used as tissue engineering materials for applications such as vascular grafts with good prospects, and their clinical application potential will be further explored in future research.

## Introduction

Most cells and extracellular matrices in human tissue possess highly ordered structures (Ellinger, 2013; Wang et al., 2018). Cells in typical tissue, including nerves, muscles, tendons, and blood vessel tissue, all exhibit oriented structural arrangements. For nerves, nerve cells are arranged in an orderly manner along the direction of the microsulcus (Tian et al., 2004; Sperling et al., 2017; Xia et al., 2022), and skeletal muscle consists of bundled muscle fibers (Zhuang et al., 2013; Ozasa et al., 2018). As regards blood vessels, endothelial cells are arranged in an orderly manner parallel to the direction of blood flow (Chen et al., 2018; Rosa et al., 2022), while smooth muscle cells are arranged along the perivascular direction (Shudo et al., 2019). Grasl et al. (Grasl et al., 2021) tried to use thermoplastic polyurethane (PUR) and polylactic acid (PLLA) to prepare axial, circumferential, and randomly arranged fiber prostheses by electrospinning and found that different fiber structures had different mechanical properties, and even vascular prostheses with good mechanical properties comparable to those of natural blood vessels could be obtained by adjusting the fiber structure. A vascular graft requires good antiplatelet properties, and the materials must possess a good antifouling ability (Yang Lei et al., 2021; He et al., 2022). Therefore, materials are required to have proper hydrophilicity.

Fibrous scaffolds prepared by electrospinning are capable of mimicking the complex structure of a natural extracellular matrix (Hasan et al., 2014; Ingavle and Kent, 2014; Kishan and Hernandez, 2017; Zong et al., 2018; Eilenberg et al., 2020), and they have large specific surface areas and a high-porosity microstructures that facilitate cell and nutrient infiltration (Yin et al., 2020a; Guo et al., 2022). The method of coaxial electrospinning can prepare functional fiber membranes (Ezhilarasu et al., 2019; Johnson et al., 2021; Zhang et al., 2021). Materials can be loaded inside the fiber to enhance the mechanical properties or regulate material degradation. Zhu et al. (Kuang et al., 2020) prepared a small-caliber vascular graft with poly (l-lactide-co-ε-caprolactone) (PLCL) as the core layer and heparin/silk gel as the shell layer. Animal experiment results showed that the graft was biodegradable and safe, it maintained a support capacity for more than 8 months, and the PLCL provided good mechanical support in the process of vascular reconstruction, promoting tissue regeneration *in vivo*. In addition, functional molecules such as growth factors or active substances could be loaded inside the fibers, allowing the active substances in the material to have a better regulatory effect through continuous and slow release. Han et al. (Han et al., 2021) prepared a functional graft loading with puerarin to promote endothelial cell proliferation and differentiation, in which the puerarin with an antiplatelet aggregation effect was used as core layer of the fibers, and Gel/PLLA was used as fiber shell layer. The results showed that puerarin could achieve long-term efficient release, the material had good mechanical properties and degradability, and the biocompatibility results indicated that the core–shell fiber prepared in this way had no cytotoxicity to endothelial cells and did not cause hemolysis.

In recent years, PLCL has been widely studied for the regeneration of soft tissue, such as blood vessels, tendons, skin, esophageal tissue, and heart tissue, due to its good biocompatibility, controlled degradation, and excellent flexibility (Jang et al., 2018; Wang et al., 2022). Blood vessels require a degree of strength to withstand high blood pressure. Research revealed that PLCL is likely suitable for use in certain vascular preparations (Kim et al., 2019), and PLCL has been approved by the United States Food and Drug Administration (FDA) for clinical applications due to its excellent mechanical properties and compliance with natural blood vessels. PLCL has been extensively studied in biomedical materials. For example, Jin et al. (Jin et al., 2019) used PLCL, silk fibroin (SF), and heparin (Hep) to prepare a double-layer vascular graft with a dense inner layer and a loose outer layer by electrospinning, which not only showed excellent performances in terms of its hydrophilicity, mechanical properties, and biocompatibility but also maintained lumen patency for 3 months after rabbit carotid artery transplantation.

Sodium tanshinone IIA-sulfonate (STS) (Cheng et al., 2018) is a water-soluble active ingredient obtained by sulfonation of tanshinone IIA extracted from the traditional Chinese medicine substance Salvia. It has been shown not only to have antithrombosis, antiplatelet adhesion, and anticoagulant functions but also to effectively improve the inflammatory response in the body, accelerate endothelial formation, and promote the formation of new tissue (Mao et al., 2006; Zhou et al., 2018; Chen et al., 2021; Zhou et al., 2021; Li et al., 2022). In previous studies, STS was usually used as an injection to treat diseases such as atherosclerosis. Zhang et al. (Zhang et al., 2022) randomly grouped mice and then injected them with STS and other control drugs. The postoperative results showed that the cardiac function of the mice in the STS group was significantly improved, and the coronary artery occlusion situation was improved, which also proved that STS had excellent anti-atherosclerosis and antiplatelet aggregation effects.

Polyethylene oxide (PEO) has good hydrophilicity and biocompatibility that can enhance the hydrophilicity of PLCL fiber membrane (Basu et al., 2017). For coaxial electrospinning, aqueous solution alone as the core layer was difficult to sustain smooth spinning during coaxial electrospinning, PEO could be applied as a stabilizer improving the viscosity of the solution, and the core layer solution would obtain suitable surface tension and achieve stable continuous spinning (Chen et al., 2015). According to the literature, PEO would be dissolved in water to improve the porosity of the fiber membrane and then enhanced the migration and proliferation ability of cells within the fiber membrane (Wang et al., 2014).

Thousands of surgeries are performed every day worldwide to cope with physical illness or injury. Tissue engineering involves a combination of autologous cells and tissue engineering materials to guide new tissue regeneration and repair or rebuild damaged tissue (O'Brien, 2011). In the process of tissue reconstruction, materials must meet the requirements of the physical and chemical properties, such as mechanical, hydrophilicity, degradation, and biocompatibility properties. PLCL has an excellent mechanical strength. PLCL of different molecular weights can achieve controllable degradation of tissue materials, and its biocompatibility can make cells attach to materials, which is conducive to the regeneration of new tissue. Considering that the orderly arrangement of cells is conducive to the connection between tissue engineering materials and natural tissue, this idea can be realized by designing tissue engineering materials with oriented structures. However, platelets are prone to accumulate in large quantities in PLCL membranes, so STS, with an antiplatelet ability, was added. The structure will affect the performance. Oriented and random structures impact the mechanical properties of the material, what could impact the material suture strength, hydrophobicity, degradation, and biocompatibility. This was explored in this study based on the above considerations.

In this study, the STS was selected as a core layer, which was wrapped with PEO, and the biocompatibility of the functional fiber membranes was expected to be improved by the addition of STS. By adjusting the orientations of the fibers and varying several parameters, such as the speed of the roller shaft at the receiving end, random- and oriented-fiber membranes were prepared with a core–shell structure, and then the properties of the fiber membranes were tentatively explored, including the mechanical properties, degradation, and hydrophilicity. In addition, the biocompatibility and hemocompatibility of the fiber membranes containing STS were analyzed.

## Materials and methods

### Materials

Poly (l–lactide-co-ε-caprolactone) (PLCL (50:50), with 50 mol% l-lactide, M_W_: 330,000 Da) was provided by Jinan Daigang (Jinan, China). 1,1,1,3,3,3-Hexafluoro-2-isopropanol (HFIP) was obtained from Shanghai Macklin Biochemical Technology (Shanghai, China). 3-(4,5-dimethylthiazole-2-yl)-2,5-dibenzotriazole ammonium bromide (MTT) was acquired from Beijing Biotopped Technology (Beijing, China). A Cell Counting Kit-8 (CCK-8) was purchased from Aladdin Reagents (Shanghai, China). Sodium tanshinone IIA sulfonate (STS) was provided by Dalian Meilun Biotechnology (Dalian, China). Poly-ethylene oxide (PEO, M_W_: 100,000 Da) was purchased from Shanghai Macklin Biochemical Technology (Shanghai, China). All reagents used for cell culture were purchased from Gibco Life Technologies (Shanghai, China) unless stated otherwise. All cells were purchased from iCell (SAIBAIKANG, Shanghai, China).

### Coaxial electrospinning for preparing functional fiber membranes and tube grafts

PLCL was dissolved in HFIP at a concentration of 10% (W/V) under room temperature and stirred for 24 h (84-1, Shanghai Meiyingpu Instrumentation Manufacturing Co., Ltd. Shanghai, China) to obtain core–shell structured fibers, with PLCL as the shell and PEO as the core. In this study, to obtain more core–shell structured fibers and to determine the physical properties of these fibers, PEO [6%, (w/v)], as a temporary substitute for various water-soluble functional substances, was used as the core material, while specific active ingredients (STS) will be added during the biological testing. For coaxial electrospinning, the PLCL solution was loaded into a 20-mL syringe with a 15-gauge needle at an injection speed of 1.3 mL h^−1^, and the PEO solution was loaded into a 10-mL syringe with a 19-gauge needle at an injection speed of .8 mL h^−1^. The needle tip was subjected to positive voltage at 11.5 kV and negative voltage at 2.5 kV (TK129, Shanghai Taco Company, Shanghai, China). By adjusting the fiber collector roller speed, random and oriented fiber membranes and tube grafts were fabricated.

### Morphology characterization of functional fiber membranes

Fiber morphology was observed using scanning electron microscopy (SEM, Apreo S, Thermo Fisher Scientific Technologies, Massachusetts, US), and the fiber diameter distribution was analyzed with Image-J software. The internal structure of the coaxial electrospinning fibers was observed through transmission electron microscopy (TEM, Talos F200X, Thermo Fisher Scientific Technologies, Massachusetts, US).

### Mechanical properties

For mechanical property testing, the tensile strength of the fiber membranes was tested, and the suture retention strength of the tube grafts also was measured by means of a universal tensile extensibility instrument (UTM2503, Shenzhen Sansi Zongheng Technology Co., Ltd., Shenzhen, China). The fiber membranes were cut into dumbbell-shaped samples with an effective width of 4 mm by punching, and the thickness of each sample was measured. Before testing, the two ends of the sample were clamped, a sensor with a load capacity of 50 N was used, and the tensile extensibility instrument—with a fixture spacing of 3.5 cm—was stretched longitudinally until it ruptured at a speed of 5 mm min^−1^. The prepared tube grafts were cut into 1-cm sections, and then the thickness of graft well was measured. Before testing, one end of the sample was clamped to one arm of the equipment and the other end, with 5–0 polyester suture (Shanghai Pudong Jinhuan Medical Products Co., Ltd., Shanghai, China) placed 2 mm from the edge, was clamped to the other arm of the equipment at a speed of 120 mm min^−1^ until rupture (UTM2503, Shenzhen Sansi Zongheng Technology Co., Ltd. Shenzhen, China).

### Contact angle

Fiber membranes were set into glass slides, and to test the hydrophilicity, a video optical contact angle measuring instrument (DSA30, Kruss, Germany) was applied. A total of 2 μL of water was dropped on a flat place of the fiber membrane to avoid air bubble formation when placing samples on glass slides, and then the video, image, and contact angle data were recorded using the instrument. After the test, the data were compiled and plotted to analyze the hydrophilicity of the membranes, which affects cell attachment and platelet adhesion.

### Material degradation behavior

The prepared samples were first sterilized and then placed in a 5-mL EP tube. A total of 3 mL PBS and 60 μL antibiotic-antimycotic solution were then added to the EP tube, after which they were placed into a constant temperature shaker (ZH-S) at 37°C to simulate the *in vivo* environment. Every week, all of the supernatant was removed and replaced with an equal volume of fresh medium, and the pH value of the supernatant was measured using a pH meter (FE28, Shanghai Mettler Toledo Instrument Co., Ltd., Shanghai, China). Every month, the samples were removed and freeze-dried (SCIENTZ-18N, Ningbo Xinzhi Biotechnology Co., Ltd., Ningbo, China), and then the samples were weighed with an electronic balance (BSA124S, Sartorius Scientific Instruments (Beijing) Co., Ltd.). The fiber membrane microstructure was also then observed using SEM (Apreo S, Thermo Fisher Scientific Technologies, Massachusetts, US).

### Blood compatibility

In the blood compatibility experiment, two methods were applied: a lactate dehydrogenase (LDH) activity assay and SEM morphology observation (S-4800, Japanese High-tech Co., Ltd.). Platelets obtained after centrifugation were used for LDH detection, and rabbit whole blood was used for SEM observation. First, the prepared sample was placed into a 24-well plate. For LDH testing, rabbit blood (purchased by Nanjing Semberga Biotechnology Co., Ltd., Nanjing, China) was centrifuged (TGL-15B, Shanghai Anting Scientific Instrument Factory, Shanghai, China) at 2,500 rpm for 10 min to obtain platelet rich plasma (prp). After that, 500 μL prp was added to each sample, which were then shaken at 37°C for 3 h, and then the samples were washed with PBS, after which 1 mL of 2% Triton was added to each. After shaking for 20 min, we extracted the solution and then centrifuged it for 10 min, taking the supernatant for further testing with an LDH release kit according to the manufacturer’s instructions. We recorded the absorbance of the reaction solution at 450 nm on a fully automatic microplate reader (DNM-9606, Beijing Pulun Xin Technology Co., Ltd. Beijing, China) for the relative quantification of platelets.

For SEM observation, 1 mL of rabbit blood was added to each sample and shaken at 37°C for 2 h. After that, the samples were washed gently with sterile water, and then 1 mL of 2.5% glutaraldehyde solution was added to each well and allowed to sit for 1 h, following dehydration with a gradient alcohol solution (30%, 50%, 70%, 80%, 90%, 95%, and 100%). Finally, the processed sample was placed in a fume hood and blow-dried, and was then immediately sprayed with gold and was observed using a scanning electron microscope.

### Cell compatibility

The vacuum-dried functional fiber membranes were punched to the same size discs and placed into a 24-well plate, and then cell experiments were performed for 1, 3, and 5 days. Samples were first sterilized under alcohol vapor for 4 h, followed by rinsing with PBS twice, and mouse fibroblasts (3T3) were then seeded at a concentration of 1×10^4^ cells•well^−1^. At the corresponding setting time point, the medium was extracted, and 40 μL MTT and 360 μL DMEM medium were then added, incubating with the samples for 4 h. After that, the supernatant was aspirated by adding 200 μL DMSO to dissolve the sediment for 20 min in the shaker. At last, 100 μL supernatant was placed into a 96-well plate for further analysis with a microplate reader at 492 nm. Absorbance was found to have a good linear relationship with the number of viable cells.

The cell seeding procedures for human umbilical artery smooth muscle cells (HUASMCs) and macrophages (RAW264.7) were same as those for the 3T3 cells mentioned above, with the only difference being the number of RAW264.7 were seeded (8×10^3^ cells•well^−1^). At the specific time point, 40 μL of CCK-8 was added to every well, and then allowed to incubate for 4 h. Finally, the supernatant (100 μL) was placed into 96-well plate for further analysis with a microplate reader at 450 nm.

After cells were cultured on different fiber membranes for 5 days, the cell morphologies were observed using a laser scanning confocal microscope. The cell membranes were washed with PBS. A total of 200 μL of 2.5% glutaraldehyde fixation was then added, and after 1 h the cells were washed 3 times with PBS, after which the cell membranes were treated with .1% Triton X-100 (PBS) for 3 min and then washed again with PBS. Then cell-membranes were blocked with 1% BSA at 37°C for 80 min, followed by washing with PBS. Finally, 200 μL of the pre-prepared Rhodamine B and DAPI solution were added to each well. After 30 min of incubation, the cell-membrane was washed in preparation for forward laser scanning confocal microscopy observation (LSM 880 with fast AiryScan, Carl Zeiss AG, Dresden, Germany).

### Statistical analysis

All quantitative results were obtained at least three samples. Statistical ANOVA analysis was performed in Origin Pro 2018 software. Results were expressed as the mean ± standard deviation (SD), with significant differences at *p < .05, **p < .01, ***p < .001.

## Results and discussion

### Microstructure

**FIGURE 1:**
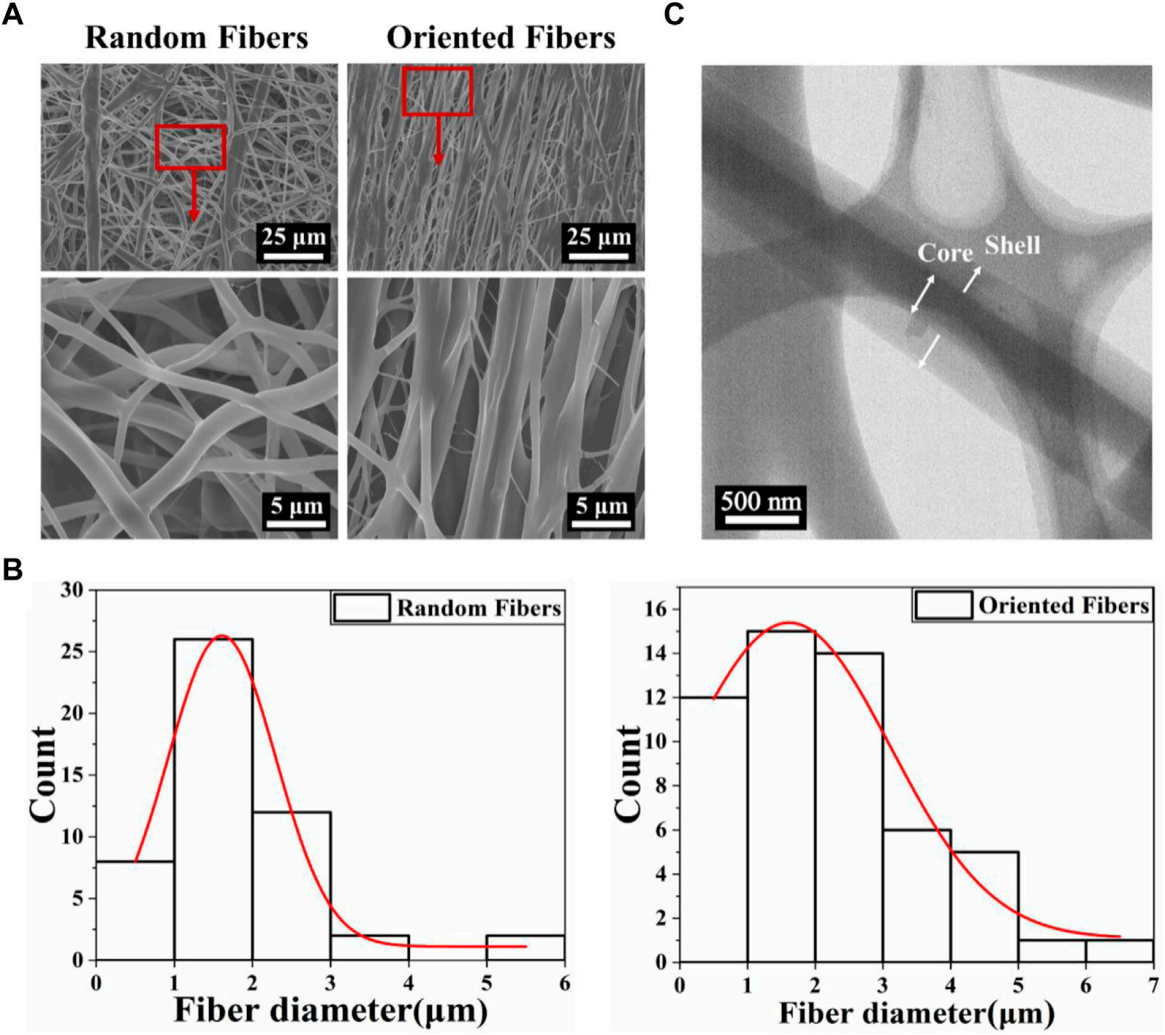
The morphology and diameter distribution of fibers with a core–shell structure. **(A)** Scanning electron microscopy images of random- and oriented-fiber membranes. **(B)** Diameter distribution of random and oriented fibers shown in relative lines. **(C)** Transmission electron microscopy morphology of fibers with a core–shell structure.

Scanning electron microscopy (SEM) images of the random- and oriented-fiber membranes are shown in Figure 1A. The images clearly show the differences between the random and oriented fibers; Figure 1B shows that the diameters of random fibers were about 1.79 ± 1.03 μm, the thickness was relatively uniform, and the distribution was random in all directions. The diameters of the oriented fibers were about 2.14 ± 1.40 μm, which were slightly larger than those of the random fibers, and most of the fibers were arranged in the same direction. Overall, all the fibers were connected without any beads or gross defects. The different arrangements of the fibers likely provided different physical, mechanical, and biocompatibility properties (Dehghan and Khajeh Mehrizi, 2022; Ozdemir et al., 2022; Yuan et al., 2022). The subsequent experiments were conducted with random- and oriented-fiber membranes, and it was expected that the oriented-fiber membranes would be more helpful to the growth and proliferation of cells and promote the regeneration of new tissue.

Figure 1C shows the transmission electron microscopy (TEM) morphology of the coaxial electrospun fiber. It is clear that the core–shell structure was successfully obtained, demonstrating that the PEO was successfully wrapped into the PLCL by coaxial electrospinning. Thus, the functional composition could be loaded inside or outside the fibers, which could improve drug delivery systems with sustained release, enhance the hydrophilicity and biocompatibility, likely reduce the rejection reaction between the fiber graft and human tissue, and improve the cell attachment. In addition, the oriented-fiber membranes containing STS are expected to be able not only to provide sustained release of STS over time and prevent platelet adhesion but also to induce cells to arrange orderly.

### Mechanical properties

**FIGURE 2:**
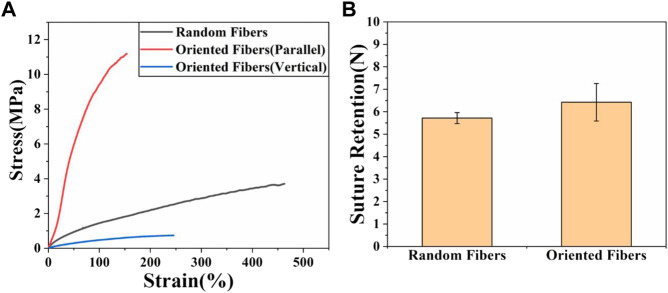
Mechanical properties of random- and oriented-fiber membranes. **(A)** Stress–strain of random- and oriented-fiber membranes parallel and perpendicular to fiber direction, respectively, at a tensile speed of 5 mm min^−1^ (N = 4). **(B)** Suture retention of random- and oriented-fiber tube grafts at a tensile speed of 120 mm min^−1^ (N = 3).

The excellent characteristics of PLCL provide it with great potential to be applied for vascular grafts, and the mechanical properties are among the most important properties for grafts. The tensile strengths of fiber membranes and the suture retention of tube grafts were tested. Figure 2A shows that the maximum stress of the random-fiber membranes was nearly 4 MPa, while that for the oriented-fiber membranes parallel to the fiber direction could reach more than 11 MPa, which was much higher than that of the random-fiber membranes. The corresponding strain for oriented-fiber membranes was 190%, which was lower than that of the random-fiber membranes (470%). This phenomenon may be explained as the orientation fibers being stretched during the spinning process such that the orientation stress was enhanced in the parallel direction. The stress for the oriented-fiber membranes perpendicular to the fiber direction was as low as .8 MPa. This was because the fibers with high order had a lower tangle degree between the fibers than that of the knotted random fibers. According to the literature, the stress range of the human aorta is .2–1.6 MPa (Famaey et al., 2014; Backman et al., 2017; Maleki and Kalantarnia, 2022). The stresses of the fiber membranes were much higher than the stress requirements of the human aorta, indicating that they met the mechanical strength requirements for implantation in the body. Therefore, the excellent mechanical properties of the fiber membranes could be applied for blood vessel grafts and good mechanical support promote the smooth flow of blood in vascular grafts, without causing an aneurysm.

The suture retention strength is very important for vascular grafts, and it should be sufficient for use in a surgical procedure. The result for suture retention testing is shown in Figure 2B. It was found that the suture retention strength of the oriented-fiber tube grafts was higher than that of random-fiber tube grafts. Overall, both tube grafts had good suture retention strengths of 5 N or higher. Based on study (Yin et al., 2020b), the suture force of animal autologous vascular tissue is 1.7 N. Therefore, both materials were completely comparable to animal autologous vascular tissue in terms of mechanical strength, which could ensure that rupture and bleeding would not occur when implanted in the body, and they could be safely used in the body.

### Contact angle

**FIGURE 3:**
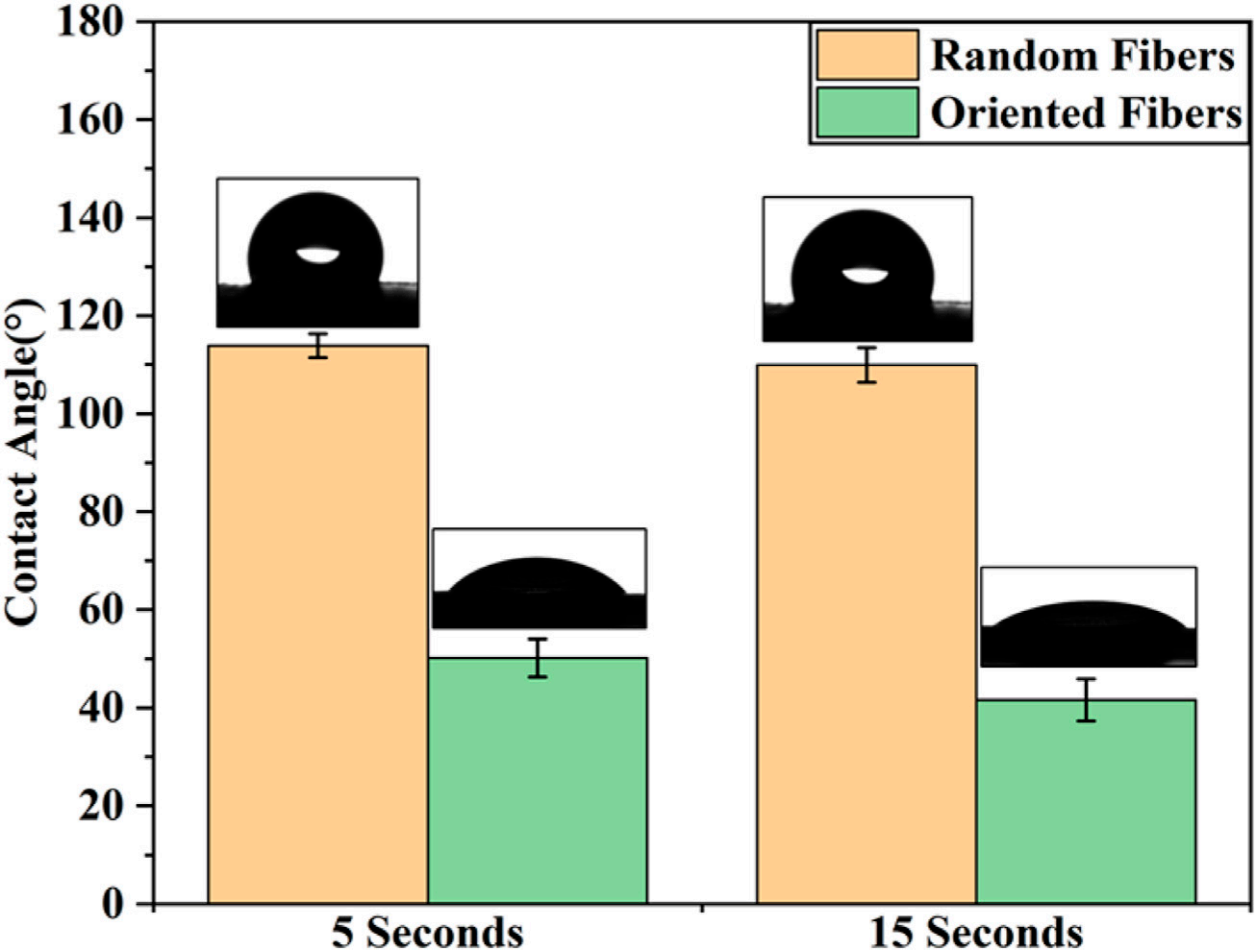
Contact angle of random- and oriented-fiber membranes at different time points (N = 5).

According to previous research (Ehtesabi, 2021; Manivasagam and Ketul, 2021; Al-Azzam and Alazzam, 2022), the graft surface hydrophilicity is closely related to the adhesion and cohesion of platelets, and the hydrophilicity can also affect the migration and proliferation of cells. In this study, whether the fiber structure with random and oriented fibers affected the hydrophobicity, hemocompatibility, and cell attachment ability were explored in follow-up testing. The hydrophilicity of the random- and oriented-fiber membranes were measured by contact angle testing, and results are shown in Figure 3. The contact angles of the random-fiber membranes were larger than those of the oriented-fiber membrane. The main reason was that the distribution of random fibers was chaotic and staggered, and water molecules could not easily penetrate the material. In contrast, the oriented-fiber membranes obtained smaller contact angles due to the high degree of fiber order. When the water molecules contacted the fibers, they quickly penetrate through the fiber gaps. According to the data, the membrane contact angles were smaller after 15 s than after 5 s, and water molecules could slowly penetrate the fibers over time. Therefore, a hydrophobic fiber membrane could be shifted to relatively hydrophilicity through microstructural changes, which is likely induce cell migration, growth, and proliferation.

### *In vitro* degradability

**FIGURE 4:**
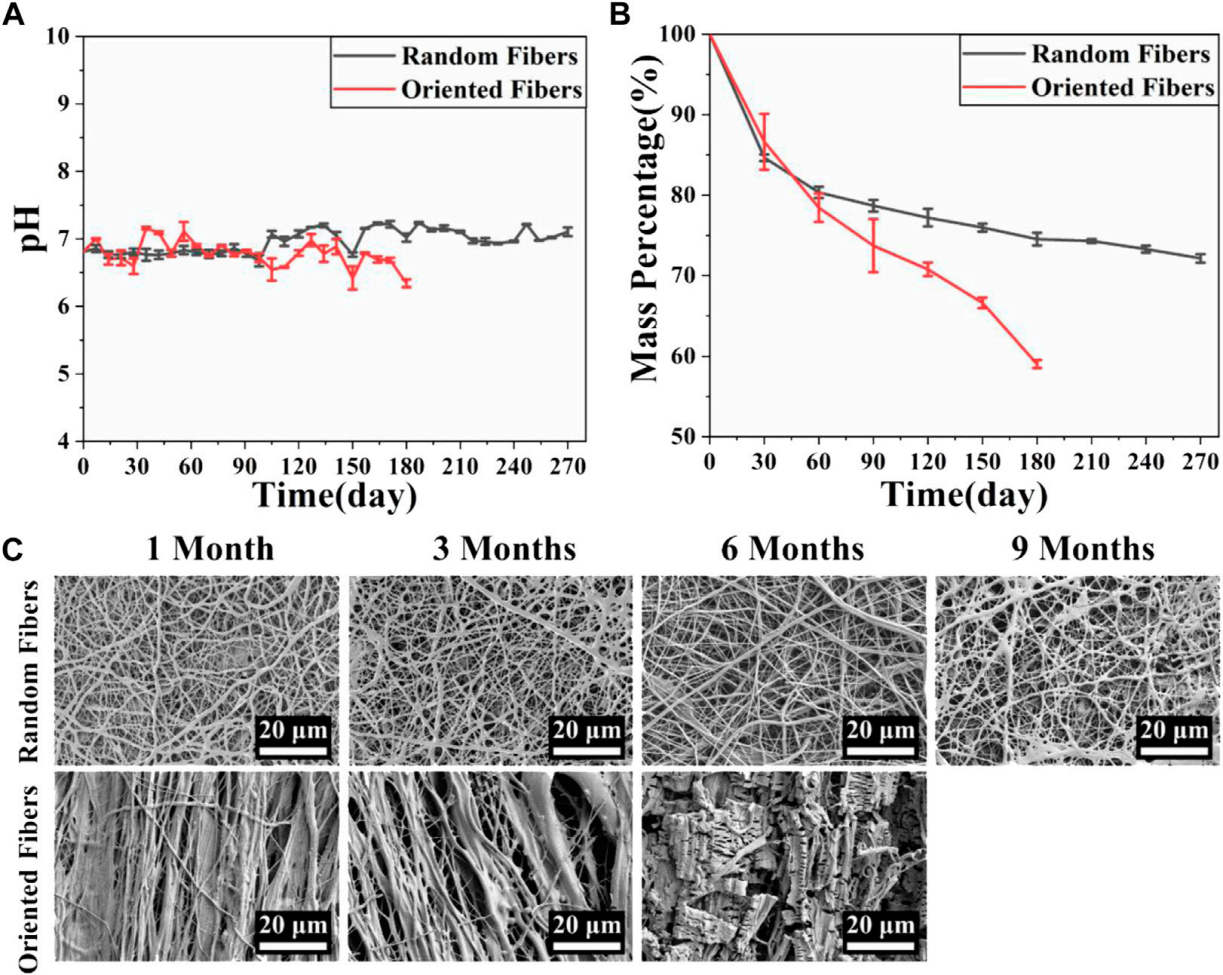
Simulation of fiber membrane degradation behavior *in vitro*. **(A)** pH changes during simulated degradation of random- and oriented-fiber membranes *in vitro* (N = 3). **(B)** Mass changes during simulated degradation of random- and oriented-fiber membranes *in vitro* (N = 3). **(C)** Morphological changes during simulated degradation of random- and oriented-fiber membranes *in vitro*.

The degradation behaviors of the membranes are closely related to the membranes’ physical and chemical properties, and appropriate degradation is important, especially for implanted grafts, which could match the tissue regeneration rate (Wang et al., 2020; Alamán-Díez et al., 2022). In this experiment, the pH and mass changes of the membranes at various time points were examined, and the microscopic morphological changes in the membranes were observed every month. Figure 4A shows the pH changes in the random- and oriented-fiber membranes. The pH fluctuated slightly between 6.5 and 7.5, and thus, it remained basically stable. The pH of the oriented-fiber membranes fluctuated between 6 and 7.5, and the fluctuation range interval was larger than that of the random-fiber membranes. However, neither membrane type had strong acidity in the degradation process, which indicated that inflammatory responses during the fiber membrane grafts implanted in the body would be reduced. In this study, the PEO loaded inside the fibers released into aqueous solutions as the fibers degraded, and they always remained neutral or weakly basic in the aqueous solutions. This caused the pH to undergo only slight changes (Hu, 2000). Figure 4B shows the mass changes of the random- and oriented-fiber membranes during *in vitro* simulated degradation. The mass decreased rapidly in the first month, and it slowly declined after the first month. Overall, the mass degradation rates of the random-fiber membranes were slower than those of the oriented-fiber membranes. After 6 months, the oriented-fiber membranes had mass losses of more than 40%, while the random-fiber membranes had mass losses of about 25%. Even in the ninth month, the random-fiber membranes retained 72% of their masses.

Figure 4C shows that the fiber structures of the random-fiber membranes remained intact in the first 6 months. In the ninth month, a partial area of fiber fracture occurred, and the fiber membranes ruptured or cracked. As shown in Figure 4B, the masses were maintained at about 72% when the random-fiber membranes were broken. While the degradation rates of the oriented-fiber membranes were relatively fast, the fiber form was intact in the third month. Fiber rupture occurred in the sixth month, and the fiber morphology was not clear. As shown in Figure 4B, the masses remaining were less than 60% when the oriented-fiber membranes were broken.

The phenomenon can be explained by the results of the contact angle tests. The contact angles of the oriented-fiber membranes were smaller than those of random-fiber membranes, indicating the oriented-fiber membranes had better hydrophilicity. Thus, water molecules could more easily penetrate through the fiber gaps for the oriented-fiber membranes, and stronger interactions between the fibers and water molecules would accelerate the degradation rate. However, it can be seen from Figure 4A that the pH values of the oriented-fiber membranes were slightly lower than those of the random-fiber membranes in degradation process, indicating that weakly acidic environment also promoted the degradation of the membranes. For the random-fiber membranes, the fibers were intertwined, and the diameters of fibers were relatively evenly distributed. As a result, water had more difficulty penetrating to the insides of membranes, and therefore, the degradation for the random-fiber membranes was slow. If both random- and oriented-fiber membranes could maintain sufficient mechanical support in the early stage when implanted *in vivo*, there would be sufficient time to ensure new tissue regeneration. According to the literature (De Valence et al., 2012; Zhou et al., 2014; Wang et al., 2017; Sevostyanova et al., 2018; Yin et al., 2020b; Wen et al., 2020; Yang et al., 2021), when a graft was implanted *in vivo*, the endothelial cells gradually formed an intima, and smooth muscle cells and fibroblasts were also arranged along the axial direction of the graft, thus forming new blood vessel tissue. After 3 months, the vascular development was almost complete. Thus, the degradation rate of the oriented-fiber graft was likely suitable for the process of new tissue regeneration, while the random-fiber graft with a low degradation rate may cause the material to reside in the tissue too long, which would impede new tissue reconstitution.

### Hemocompatibility

**FIGURE 5:**
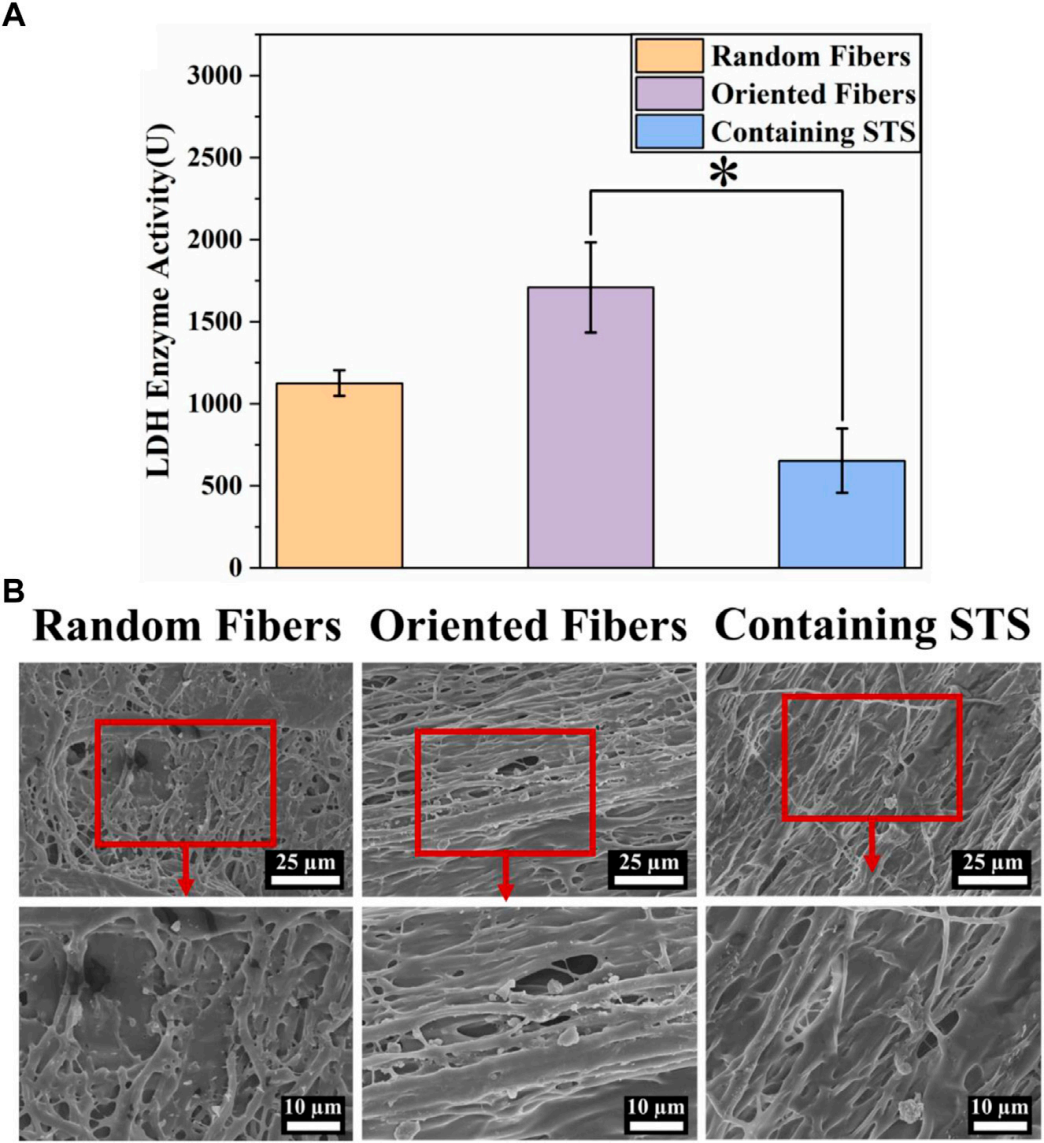
Hemocompatibility of fiber membranes. **(A)** LDH activities of random-fiber membranes, oriented-fiber membranes, and oriented-fiber membranes containing STS (N = 3, *p < .05). **(B)** Platelet adhesion on random-fiber membranes, oriented-fiber membranes, and oriented-fiber membranes containing STS.

The blood compatibility of the functional fiber graft is important, and the blood must flow smoothly when the grafts are implanted in the body. In this experiment, platelet adhesion on different membranes was analyzed by the LDH activity assay and SEM. The results in Figure 5A show that when platelet adhesion was measured by the LDH kit, the absorption values for the random-fiber membranes were slightly smaller than those of the oriented-fiber membranes. The data showed that the oriented-fiber membranes containing STS had a good antiplatelet adhesion effect, with lower absorbance than the others, and the effect was significantly different from those of the oriented-fiber membranes. The excellent antiplatelet effect likely was caused by STS, which possessed a good antithrombotic effect (Zhou et al., 2019; Li et al., 2020). Through the results of SEM observations in Figure 5B, the random- and oriented-fiber membranes without STS had more platelets, while there were fewer platelets on the oriented-fiber membranes containing STS. Overall, the platelets on the random fibers were relatively dispersed, and more plasma composite was coated on the random-fiber membranes. Platelets on the oriented-fiber membranes without STS could easily accumulate in the gaps of fibers, especially those that were highly ordered, which was mutually corroborated with the LDH result. Therefore, the oriented-fiber membranes containing STS showed good prospects for application for cardiovascular tissue repair with an antiplatelet adhesion effect.

### Cytocompatibility

**FIGURE 6:**
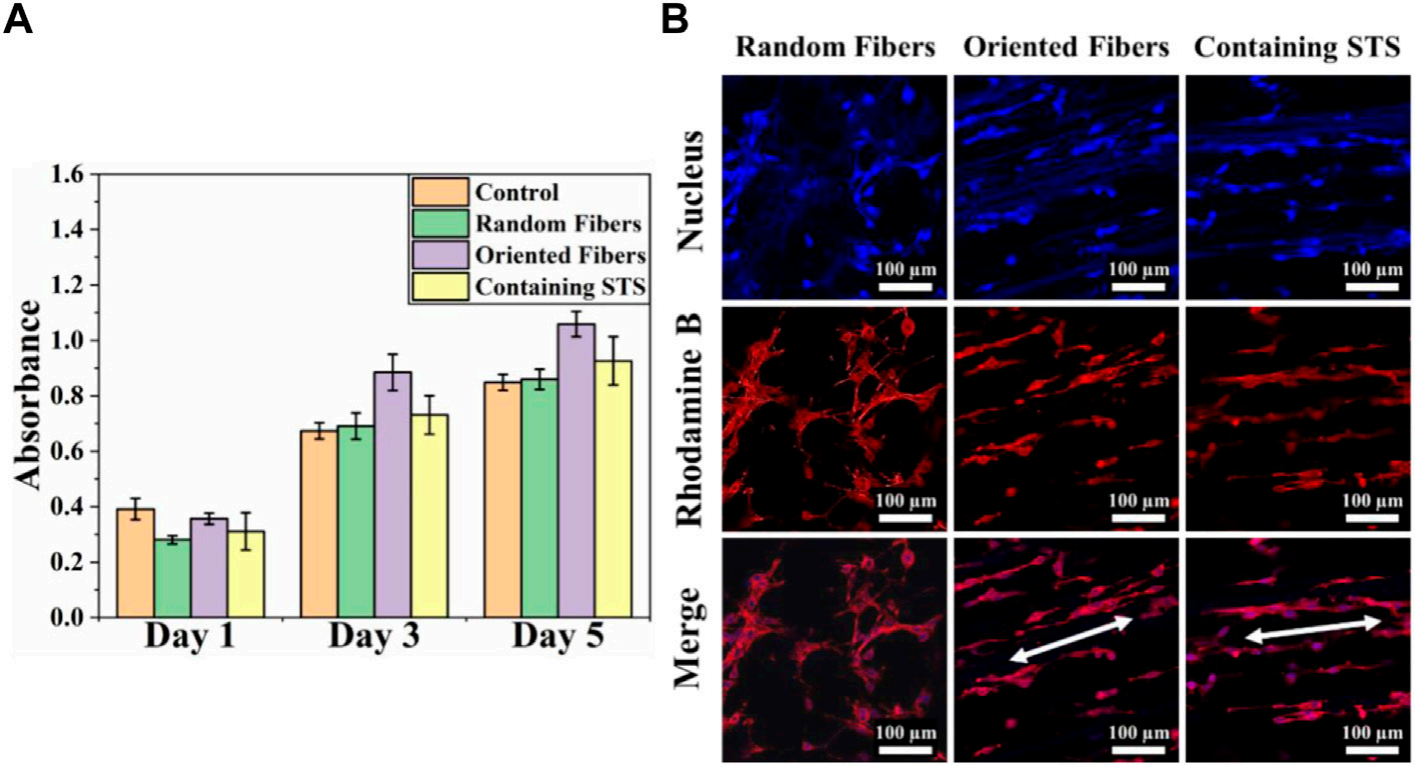
Cytotoxicity analysis of fiber membranes. **(A)** Cell compatibility of 3T3 on random-fiber membranes, oriented-fiber membranes, and oriented-fiber membranes containing STS (N = 3). **(B)** Laser scanning confocal microscopy of 3T3 on random-fiber membranes, oriented-fiber membranes, and oriented-fiber membranes containing STS.

Cytotoxicity tests can confirm whether materials can be safely in contact with tissue, and materials with low cytotoxicity can reduce the risks during implantation in the body. In this study, we analyzed the toxicities of the membranes through the MTT assay, and cell morphology was observed by laser scanning confocal microscopy. Figure 6A clearly demonstrates that 3T3 could continue to grow and proliferate on all kind of membranes, and the absorbance values for the orientated-fiber membranes exceeded those of the control slightly. Therefore, all these membranes had good cellular biocompatibility. From Figure 6B, it can be seen that 3T3 could be arranged in one direction on oriented-fiber membranes with or without STS.

**FIGURE 7:**
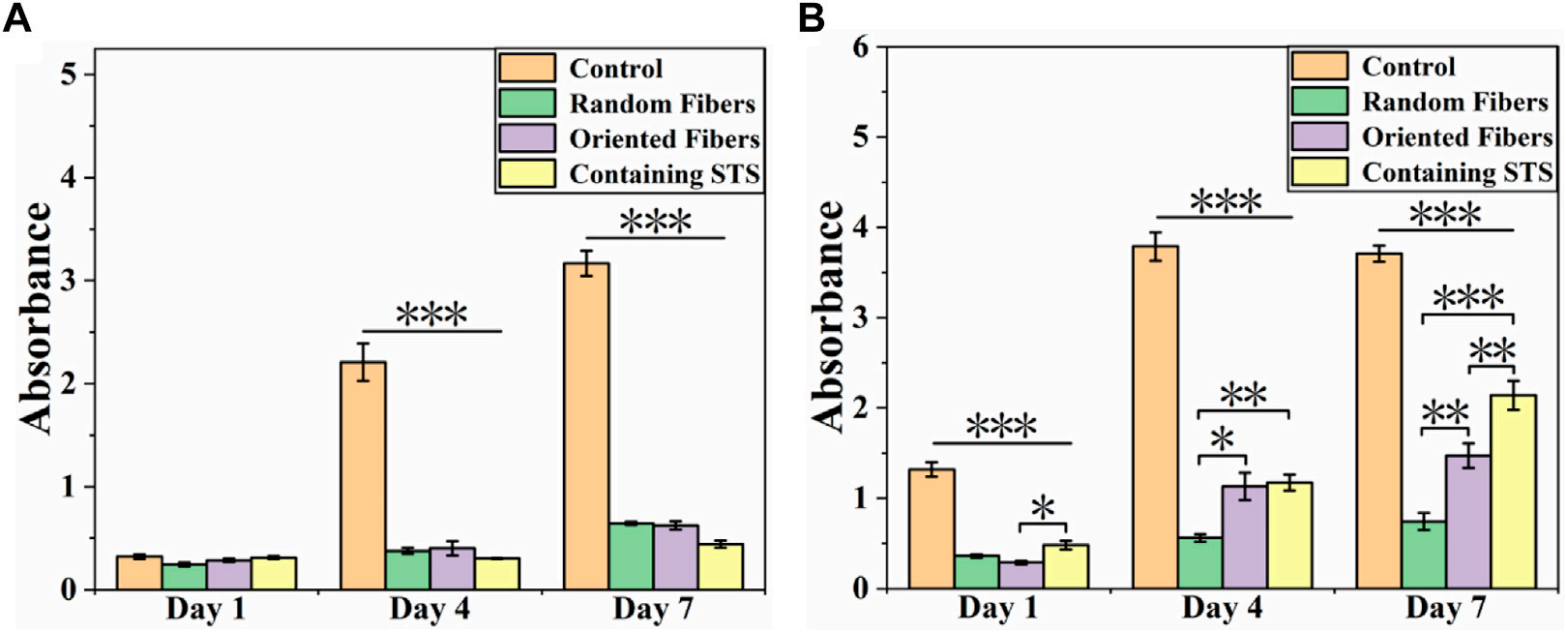
Cell compatibility of **(A)** Raw264.7 and **(B)** HUASMCs on random-fiber membranes, oriented-fiber membranes, and oriented-fiber membranes containing STS (N = 3). *** above the horizontal line indicates a significant difference between the control group and all the other groups, and *p* < .001. ***, **, and * above the parentheses indicate significant differences between the two groups under the parenthesis line, and ***p < .001, **p < .01, and **p < .05.

**FIGURE 8:**
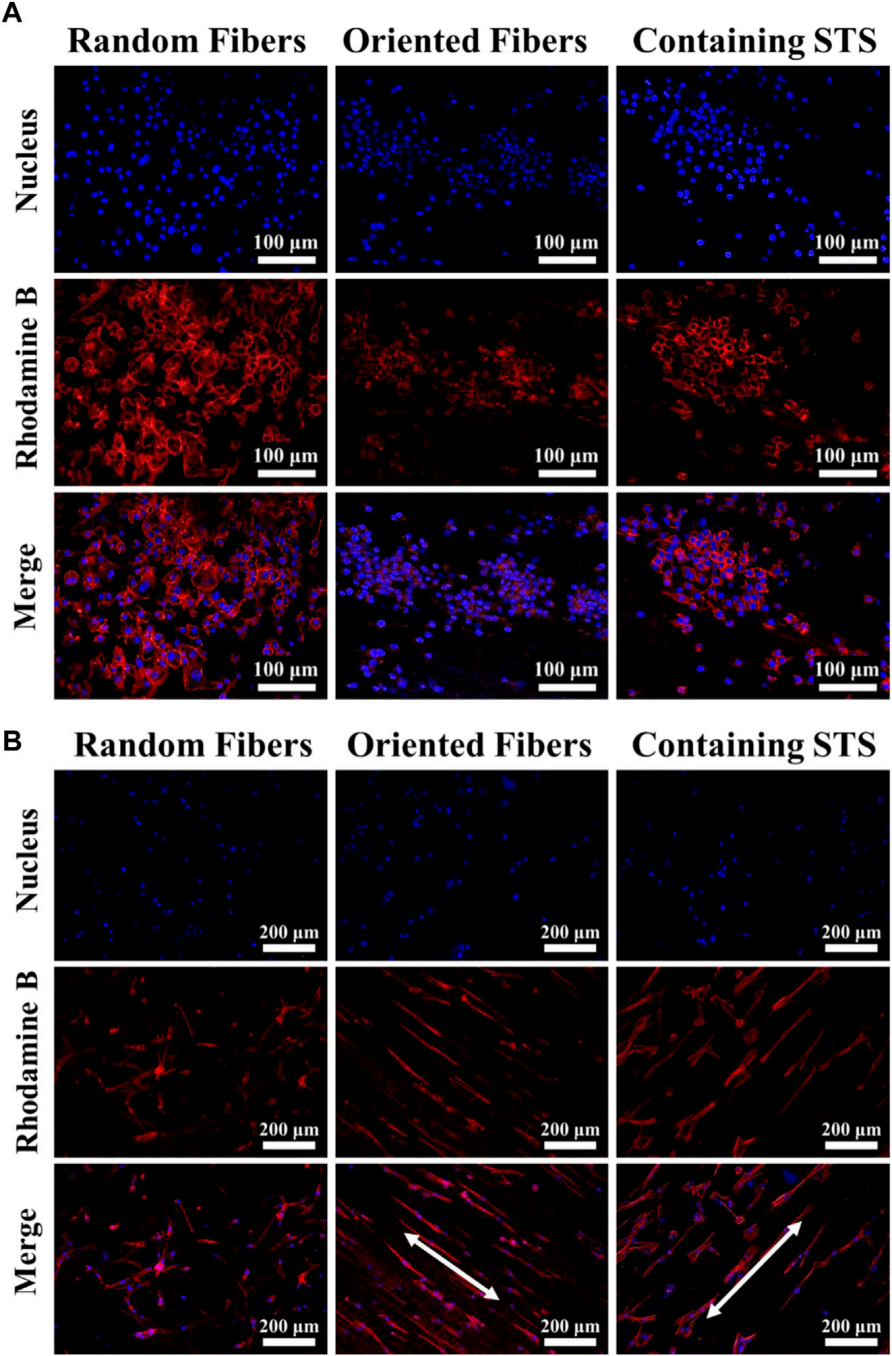
Morphologies of **(A)** RAW264.7 and **(B)** HUASMCs under laser scanning confocal microscopy on random-fiber membranes, oriented-fiber membranes, and oriented-fiber membranes containing STS.

Cell compatibility of RAW264.7 and HUASMCs on materials was tested *via* the CCK-8 assay. From Figure 7A, it can be seen that the growth of RAW264.7 in the control group was more rapid than that of the other three groups, and the cell growth statuses in the random-fiber membranes, oriented-fiber membranes, and oriented-fiber membranes containing STS were similar, without significant differences. Figure 7B showed that HUASMCs in the control group proliferated significantly faster than in the other three groups, and the HUASMCs on the three kinds of membranes had different growth rates. The random-fiber membranes had the slowest proliferation rate, and HUASMCs on the oriented-fiber membrane containing STS had the fastest proliferation rate. On day 7, cells on the oriented-fiber membrane with STS had higher proliferation than those on the other two membranes. This indicated that the oriented structure was effective to the proliferation of HUASMCs (Wang et al., 2013; Chen et al., 2017). Figure 8A shows that the growth of RAW264.7 was disordered on the random- and oriented-fiber membranes. Figure 8B shows that HUASMCs were disordered on the random-fiber membranes, while cells could be arranged in the same direction on the oriented-fiber membranes and oriented-fiber membranes containing STS. This confirmed that the oriented-fiber structure could effectively guide the arrangement of HUASMCs but not RAW264.7.

The distribution of cells on the random-fiber membranes showed an anisotropic disorderly arrangement. The behavior of 3T3 and HUASMCs was affected by the fiber microstructure, the parallel fibers induced cell migration along the fiber orientation. In summary, the membranes prepared were non-cytotoxic, and cells were in a good growth state on the membranes. Furthermore, the cells could be arranged in an orderly manner in the direction of the oriented fibers. Hence, this kind of fiber membrane, especially the oriented-fiber membranes consisting of STS, has high potential for *in vivo* implantation to repair tissue.

## Conclusion

We prepared random- and oriented-fiber membranes with fibers that had core–shell structures. PLCL fibers were the shell layer, and PEO or STS/PEO were the core layer. The fibers were prepared by coaxial electrospinning. The fiber membranes we prepared had excellent mechanical properties and suture retention strengths as well as suitable degradability. The results indicated that the introduction of PEO could improve the pH fluctuations of the membranes during *in vitro* degradation, which may be conducive to reducing the inflammatory response and the gradual rupture of the fibers. From the structural analysis, the oriented-fiber graft could theoretically promote the formation of new tissue and provide good support. The hydrophilicity was increased for the oriented-fiber membranes due to the greater amounts of aqueous solution penetrating inside the fiber membranes. Moreover, the oriented-fiber membranes with the inner layer of STS had good biocompatibility and hemocompatibility. In summary, the oriented-fiber graft was more beneficial to the formation of new tissue and provided better mechanical support than the random-fiber graft, demonstrating that the oriented-fiber membrane with STS loading with a core–shell structure had good potential for vascular graft application.

## Data Availability

The original contributions presented in the study are included in the article/Supplementary Material, further inquiries can be directed to the corresponding author.
